# Ferritin Single-Electron Transistor

**DOI:** 10.1021/acs.jpcb.4c01937

**Published:** 2024-06-25

**Authors:** Jacqueline A. Labra-Muñoz, Herre S. J. van der Zant

**Affiliations:** † Kavli Institute of Nanoscience, Delft University of Technology, 2628 CJ Delft, The Netherlands; ‡ Department of Physics, Huygens-Kamerlingh Onnes Laboratory, Leiden University, 2300 RA Leiden, The Netherlands

## Abstract

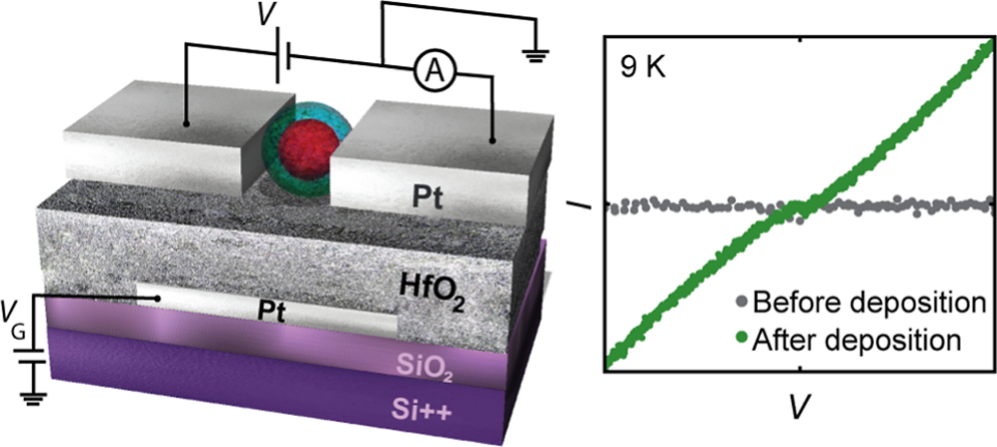

We report on the fabrication of a single-electron transistor based on ferritin using wide self-aligned nanogap devices. A local gate below the gap area enables three-terminal electrical measurements, showing the Coulomb blockade in good agreement with the single-electron tunneling theory. Comparison with this theory allows extraction of the tunnel resistances, capacitances, and gate coupling. Additionally, the data suggest the presence of two separate islands coupled in series or in parallel: information that was not possible to distinguish by using only two-terminal measurements. To interpret the charge transport features, we propose a scenario based on the established configuration structures of ferritin involving either iron sites in the organic shell or two dissimilar clusters within the core.

## Introduction

During the last three decades, the study of the electronic properties of protein structures has gained increasing interest as they are considered to be attractive elements in electronic devices due to their unique and specific physical properties (electrical^1,2^ and optical^3,4^) and chemical functions, e.g., selective binding.^5−10^ Examples of the possible use of proteins in bioelectronic devices include biomolecular transistors for data storage,^11^ biomolecular circuits,^12^ artificial retinas,^13^ early disease detectors,^14,15^ and environmental toxin sensors.^16,17^ Several experimental and theoretical research efforts have therefore been focused on understanding electron transfer via proteins through the analysis of their electronic conduction.^18−22^ The experimental approaches include the use of different scanning probe techniques (e.g., scanning tunneling microscopy and conductive atomic force microscopy) for single-protein studies and of vertical junctions with monolayers or multilayers of proteins,^21,23,24^ in which many proteins are contacted in parallel.

Regarding the integration of proteins in a three-terminal, solid-state transistor device, several attempts to use protein monolayers have been reported.^25−29^ A protein thin-film transistor of a 100 nm nanogap coated with an azurin monolayer^25^ has been fabricated; however, the conductivity deteriorated over time. Another transistor based on a protein monolayer was fabricated using bovine serum albumin, showing high gate sensitivity.^29^ Nevertheless, attempts to fabricate an electronic device using one single protein are sparse: examples include an azurin-based transistor^28^ and a transistor based on using self-assembly between an antibody and antigen to make molecular junctions with immunoglobulin G (IgG).^30^ Interestingly, the latter shows that the resistance measured in the presence (solution) or absence (vacuum) of water remained unchanged.

Reports to fabricate a single-electron transistor (SET) using a protein are even more scarce. A proof of principle experiment was performed by Li et al.^31^ in 2012, who suggested that proteins with redox centers, specifically myoglobin, located between electrodes with nanometer spacing, can be used to generate SETs at cryogenic temperatures. In some cases, the data were consistent with resonant tunneling through the heme group of single myoglobin and, in other cases, it seemed to involve two hopping steps. To our knowledge, apart from Li et al., no other SET-based device on proteins has been reported. There have been studies on SET fabrication that use proteins as a template to position nanoparticles, but the protein structure is then removed^32^ and, therefore, it is not part of the SET. Also, hybrid biodevices in which proteins have been used in addition to independently fabricated SETs were published as well.^33,34^

Previously, the fabrication of single-electron devices using ferritin^35^ particles was described by us,^36^ in which single ferritin particles were trapped in wide self-aligned nanogaps, displaying Coulomb blockade behavior. However, due to the poor gate coupling of the back gate in those devices, well-resolved three-terminal measurements could not be performed. Here, we report on the fabrication of SET-based ferritin devices, by trapping ferritin particles between wide, self-aligned electrodes that have a local gate electrode underneath the gap area, which increases the gate coupling by at least 1 order of magnitude compared to the back-gate devices. The increased gate coupling facilitates the study of the ferritin transport properties as a function of gate voltage.

## Materials and Methods

### Ferritin Sample

**Figure 1:**
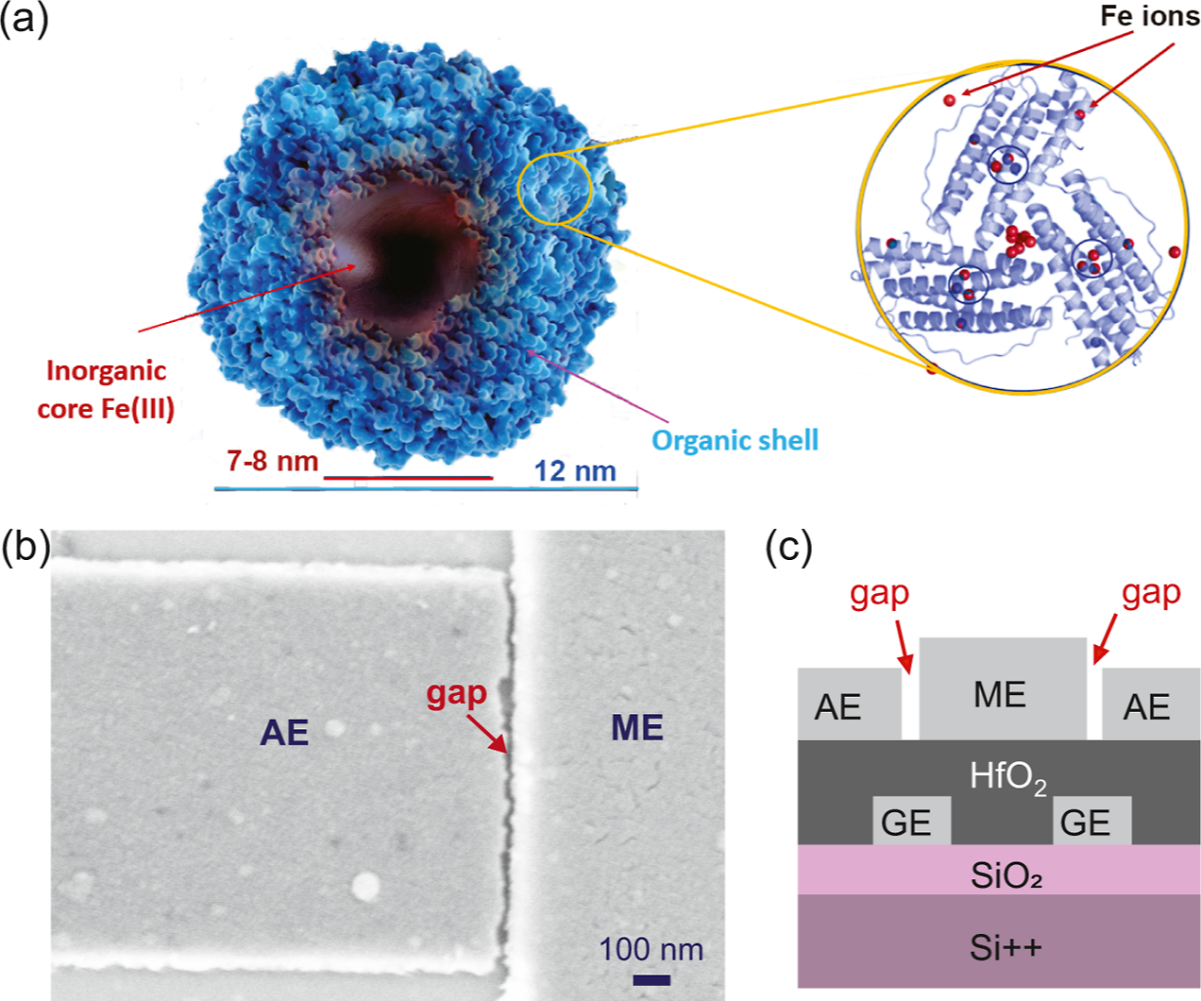
(a) Schematic representation of ferritin. The close-up of the ferritin shell that is displayed on the right side is based on ref (39) (by Chen P., licensed under Creative Commons Attribution 4.0 International) depicting redox centers in which Fe(II) can be oxidized to Fe(III). (b) Scanning electron microscopy image of an empty device showing a gap size of 8–29 nm between the source (main electrode, ME) and drain (auxiliary electrode, AE) electrodes. (c) Schematic of the lateral view of a chip displaying two gaps, one on either side of the main electrode. The local gate electrode (GE) is placed underneath both gaps.

A commercial buffer-based horse-spleen ferritin solution, purchased from Sigma-Aldrich (Cat. no. 270-40, Lot: 08E1805) of 54 mg/mL protein concentration, was used in the experiments with no further purification. The solution was diluted to a final concentration of 270 μg/mL. Figure 1a shows a schematic representation of a ferritin particle. The organic shell is represented in blue, assembled by 24 polypeptide subunits.^37^ The dark-red region in the center represents the ferritin core, which corresponds to iron stored in a mineral form (principally ferrihydrite).^37^ On the right side of the figure, a close-up of specific locations of iron Fe(II) ions in the organic shell (redox centers) is illustrated. In these redox centers the Fe(II) ions can be oxidized to Fe(III).^38^ The core’s size varies between 4 and 8.6 nm, while the organic shell is about 2 nm thick. The core-size distribution of ferritin cores was determined through transmission electron microscopy (TEM), following a previously published route.^36^

### Devices

Wide self-aligned nanogaps were fabricated following the fabrication route described in the Supporting Information (Figure S2). A chip contains one main electrode (Pt, 30 nm thick), 36 auxiliary electrodes (Pt, 20 nm thick), and a local gate electrode (Pt, 13 nm thick). The main electrode (ME) acts as the source (or drain) and one auxiliary electrode (AE) acts as the drain (source). The gate dielectric is hafnium oxide with a thickness of ∼22 nm. Figure 1b shows a scanning electron microscopy (SEM) image of a device before ferritin deposition. Panel (c) presents a schematic of the lateral view of a chip, displaying two junctions (e.g., two source-gap-drain pairs). The distance between the source and drain electrodes (gap size/length) varied between 8 and 29 nm depending on the fabricated batch. The gap width, determined by the width of the AE, is 1 μm.

### Ferritin Trapping

The procedure to investigate the ferritin trapping characterization is the same as the one described in ref (36) but performed at low temperatures. This is due to the knowledge acquired on the electrical characterization of single ferritin particles reported in that article^36^ in which similar devices are used. According to this previous characterization, at room temperature, the current vs voltage (*IV*) characteristics of ferritin are unstable (e.g., present hysteretic and switching behavior), whereas at sufficiently low temperatures, e.g., 100 K or below, the *IV*s are stable and Coulomb-blockade-like features are visible. Consequently, in this study, the inspection of ferritin trapping was performed at low temperature, rather than at room temperature.

**Figure 2:**
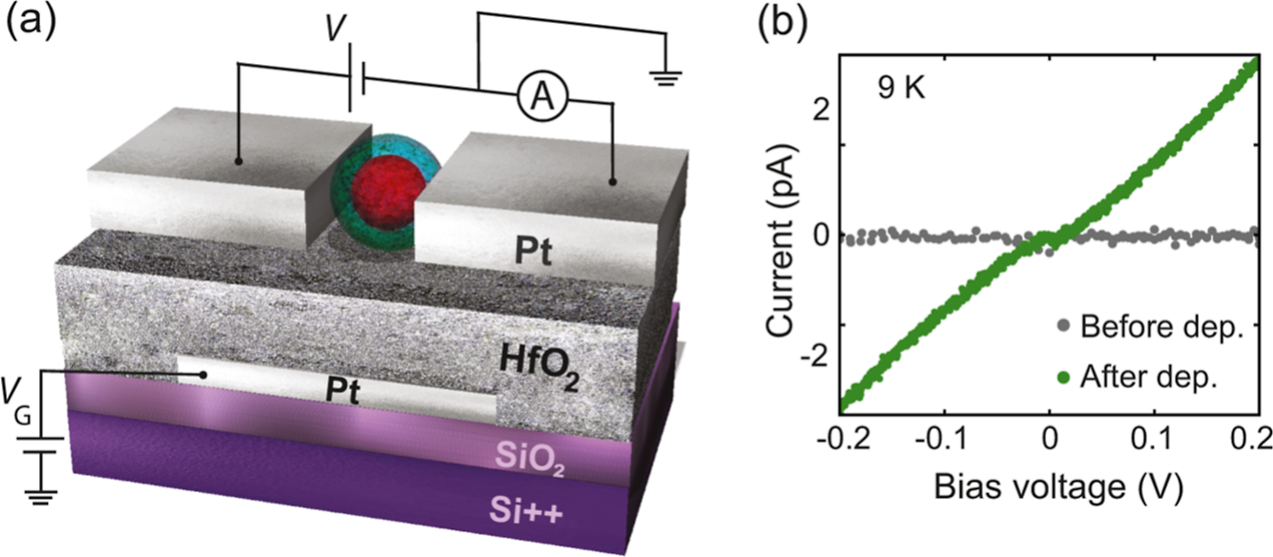
(a) Schematic circuit of a SET device with a ferritin particle. The ferritin shell and core are portrayed in green and red, respectively. A voltage source is connected to the source and drain electrodes, and the current is measured while the bias voltage (*V*) is swept from negative to positive values, and vice versa. A local gate electrode (made of Pt) is used for three-terminal characterization. (b) Electrical characterization of device Ft1 before (gray dots) and after (green dots) ferritin deposition, measured at 9 K, in vacuum, in a cryogen-free probe station. The clear increase in current after deposition indicates the successful trapping of ferritin.

Figure 2a shows a schematic of the electrical circuit. All measurements reported in this article were performed in a cryogen-free probe station and in vacuum. Before deposition, the current through the gap was measured (at room temperature) while the voltage between the source and drain electrodes (*V*) was swept from negative to positive values and vice versa. If the device was correctly fabricated, the measured *IV* should resemble that of an open circuit (gray dots in Figure 2b); on the contrary, if the current is larger than the noise floor (2 pA) over the bias voltage range probed (±200 mV), the devices were discarded.

After inspecting the bare devices, around 2–4 μL of the ferritin solution was drop-casted on top of the chip followed by immediate vacuum pumping. Afterward, the devices were cooled down (to ∼20 K or below), and the current while sweeping *V* was recorded. If a clear increase in current was detected (green dots in Figure 2b), ferritin trapping was confirmed. If no trapping was detected on any of the junctions, the chip was warmed up and another deposition took place, followed again by *IV* inspection at low temperature. A maximum of four depositions was performed for each chip. To facilitate the visual comparison between the *IV*s recorded before and after the deposition, the data displayed in Figure 2b correspond to the same temperature (9 K).

## Results

After trapping ferritin, confirmed by the observation of Coulomb-blockade-like features in the *IV* characteristics at low temperatures (green dots in Figure 2b), three-terminal electrical measurements were performed to explore the possibility of generating a single-electron ferritin transistor (SET) based on the wide self-aligned nanogaps with a local gate. A SET is a device consisting of a conductive island weakly connected to source and drain electrodes, and capacitively coupled to a (local) gate. In this case, the island is the ferritin core which is connected to the drain and source electrodes by tunnel barriers that correspond to the ferritin shell.

Two chips were subjected to three-terminal electrical measurements using the local back gate. Chip 1 had in total 25 devices that were open before deposition. After the third ferritin deposition, only one device trapped ferritin (device *#*25). We will refer to this device as device Ft1. Chip 2 had 11 devices that were open before ferritin deposition. After the first deposition, one device trapped ferritin (device *#*31); we will call it device Ft2. After finalizing the three-terminal measurements, the chip was warmed up to 70 K. It was then cooled down again, resulting in a different Coulomb diamond, which we will refer to as device Ft3. In these three devices, three-terminal electrical measurements were executed. Notice that structural changes are not expected below 100 K. However, small rotations or displacements can affect how the ferritin is coupled to the source and drain electrodes. This can lead to variations in the preferred transport path, resulting in a different Coulomb diamond.

### Closing Coulomb Diamonds

**Figure 3:**
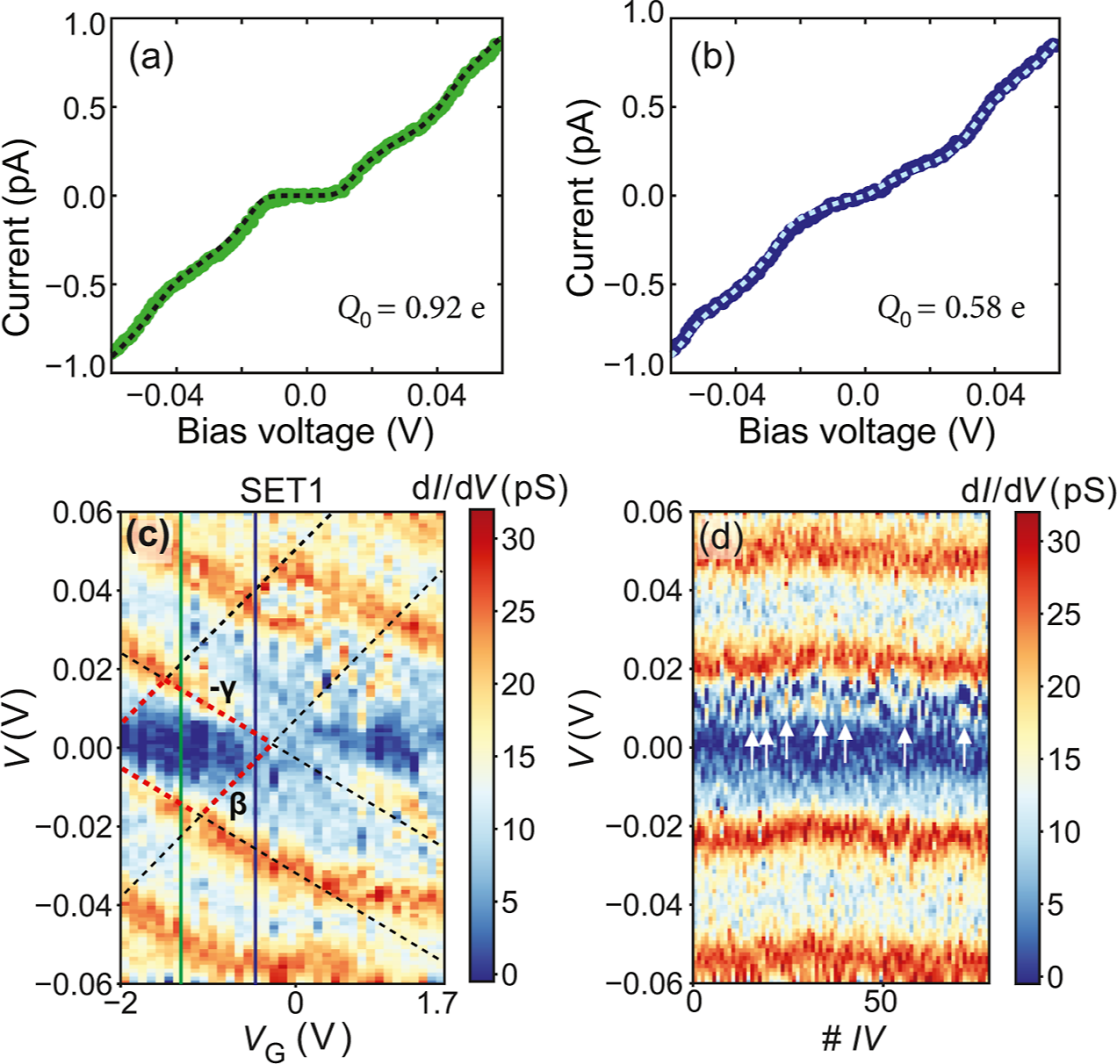
Three-terminal measurements on device Ft1, measured at 10 K. Panels (a) and (b) show two *IV*s measured at a gate voltage of −1.3 and −0.6 V, respectively. The Coulomb blockade model is fit to these curves as indicated with the dotted lines. The differential conductance of these two *IV*s is indicated by the corresponding colored-vertical lines on the stability diagram of device Ft1 [panel (c)]. This stability diagram is generated by numerical differentiation of the *IV*s recorded while sweeping *V* from negative to positive values. The slopes of the Coulomb diamond are β = 0.024 and γ = 0.015. From these slopes and the height of the diamond, α = 0.009 and *E*_C_ = 9.05 meV were estimated. *C*, *C*_1_, *C*_2_, and *C*_*G*_ are displayed in Table S1. (d) Stability diagram of d*I*/d*V* as a function of time, which is given by the *IV* number. In total, 67 consecutive *IV*s are shown; the total measurement time of the stability diagram is ∼589 min.

The gate-modulated transport through ferritin is confirmed by Figure 3 [panels (a) and (b)], where two *IV* characteristics recorded on device Ft1 are shown at two different gate voltage (*V*_G_) values. At *V*_G_ = −1.3 V [panel (a)], the *IV* presents clear Coulomb blockade steps. In contrast, at *V*_G_ = −0.6 V [panel (b)], the steps are less visible and the blockade part of the *IV* is almost linear, showing a zero-bias resistance of ∼116 GΩ (determined by a linear fit between ±20 mV), i.e., the gate has lifted the blockade. Both *IV*s were fitted to the Coulomb blockade model [dotted lines in panels (a) and (b)], following the same procedure as the one described in our previous study.^36^ The CB parameters are the capacitances between the ferritin core and the source (and drain) electrode(s) (*C*_1_, *C*_2_), the associated tunnel barrier resistances (*R*_1_, *R*_2_), the gate capacitance (*C*_G_), the offset charge (*Q*_0_), and the experimental temperature (*T*). The values that were used in the simulations are *C*_1_ = 5.75, *C*_2_ = 2.8 aF, *R*_1_ = 34, *R*_2_ = 14 GΩ, *C*_G_ = 0.1 aF, and *T* = 10 K (experimental temperature). The only difference between the two curves is the offset charge *Q*_0_ (0.92 and 0.58 e) reflecting the influence the gate has on the electrochemical potential of the island: Δ*Q*_0_ = *C*_G_Δ*V*_G_, yielding *C*_G_ = 0.4 aF. The differential conductance (d*I*/d*V*) of these two *IV*s is indicated with the corresponding colored vertical line in panel (c).

Panel (c) in Figure 3 displays the stability diagram acquired on device Ft1 presented as a colormap of the differential conductance (d*I*/d*V*) as a function of the gate voltage (*V*_G_) and the source-drain voltage (*V*). A Coulomb diamond is visible (enclosed by the red-dotted lines) and its slopes (β and γ) are used to calculate the gate coupling (α) of the device according to the relation α^–1^ = β^–1^ + γ^–1^.^40^ We find α = 0.009. Additionally, the height of the diamond (from the minimum at negative voltages to the maximum at positive bias voltages) corresponds to four charging energies (*E*_C_) with *E*_C_ = *e*^2^/2*C*, where *C* is the total capacitance of the island (*C* = *C*_1_ + *C*_2_ + *C*_G_), resulting in an estimated charging energy of ∼9.1 meV for this device. The parameters *E*_C_, *C*, *C*_1_, *C*_2_, and *C*_G_ that were obtained from the CB diamond are displayed in Table S1. The *IV*s utilized to build the colormap shown in panel (c) correspond to the ones recorded when sweeping *V* from negative to positive values. Notice that the experimental data show small switches in the d*I*/d*V*, which hinders distinguishing the diamond features. However, the noticeable agreement between the data and the Coulomb blockade model strongly indicates that the main transport is dominated by the charge transport through one ferritin particle, whose chemical potential can be controlled by varying *V*_G_. Panel (d) shows the stability diagram of recording *IV* characteristics over time at *V*_G_ = 0 V, i.e., when no gate voltage is applied. The figure demonstrates that the Coulomb blockade features (gap and steps) are stable over time. However, as in panel (c), sudden small jumps in the conductance occur. A few of these fluctuations are indicated by the white arrows.

Figure S4a shows the stability diagram recorded on device Ft2, at 10 K. Again, a Coulomb blockade diamond is visible, indicated by the black-dotted lines as a guide to the eye. From the parameters β and γ, α is estimated to be 0.01. From the height of the diamond, an estimated *E*_C_ = 12 meV is obtained. Note that the values of α and *E*_C_ are similar to those found for device Ft1.

### Nonclosing CB Diamonds

**Figure 4:**
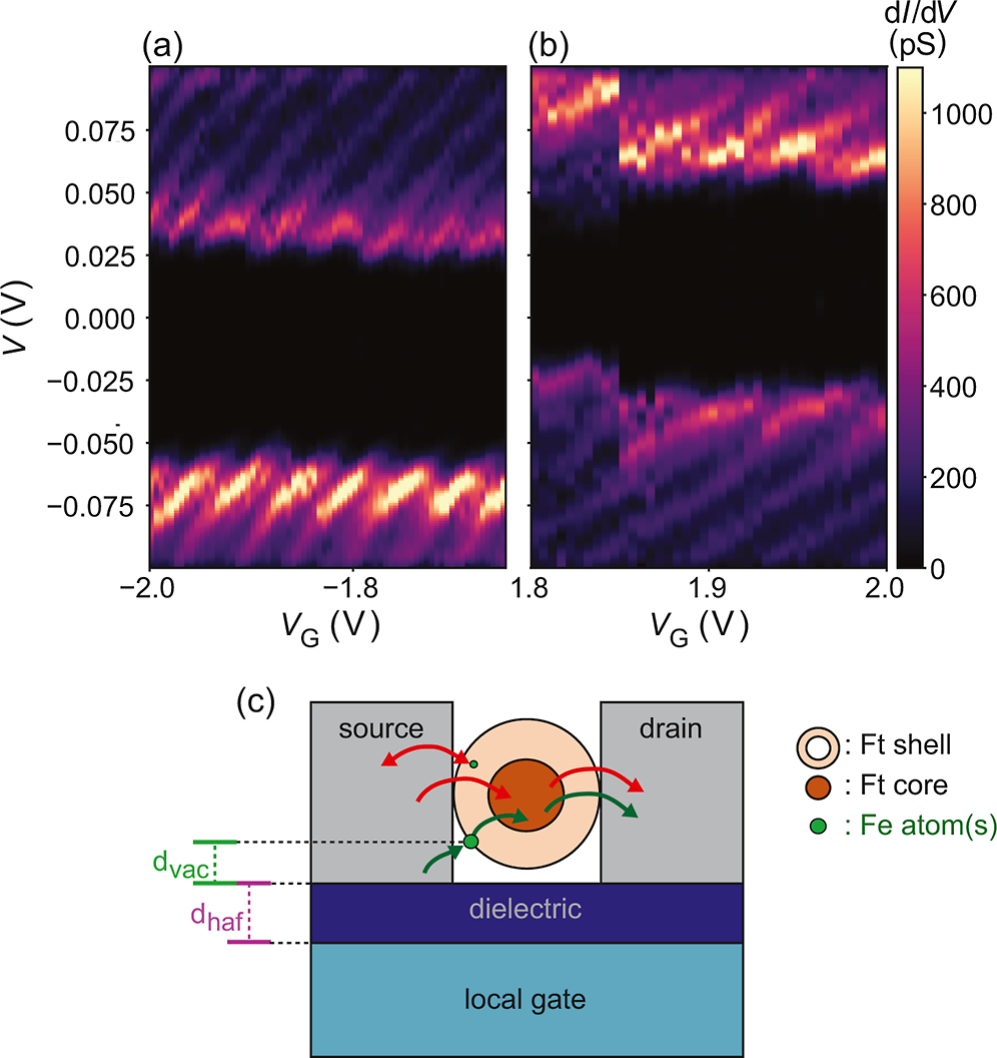
Experimental stability diagrams exhibiting nonclosing Coulomb blockade diamonds, acquired on device Ft3, at 10 K, while *V* is swept from positive to negative values. (a) At negative gate voltage range. (b) At positive gate voltage range. (c) Schematic of the capacitance of the small island (green circle), formed by one or more Fe atoms. The estimates are based on the scenario in which successive tunneling between two islands of different sizes is expected. The distance between the small island and the dielectric layer is indicated by *d*_vac_. The thickness of the dielectric (hafnium oxide) is represented by *d*_haf_.

The second distinctive Coulomb blockade-like feature that was observed in the stability diagrams of device Ft3 is a pattern of Coulomb diamonds that does not close at zero bias, which suggests the presence of two islands connected in series. An example is presented in Figure 4, where two stability diagrams acquired are included; both were measured at 10 K while sweeping the bias voltage from the maximum positive value to the minimum negative one. The shape and size of these diamond-like structures can be thought of being the superposition of one large diamond and many smaller ones. Accurately determining the parameters is challenging, but crude estimations for both can be pursued.

The first feature is the small-slanted diamond-like regular pattern that is observed in both diagrams. A guide to the eye is provided in Figure S5 by the gray-dotted lines. One of these small diamonds is indicated by the light-blue-dotted lines. From the height and slopes of this diamond, a charging energy of 7.6 meV and an α = 0.2943 were obtained. The second Coulomb diamond-like structure, seemingly of a larger energy scale, is also present: the blockade gap of this diamond is indicated by the yellow-dotted lines (Figure S5). If the length of the yellow-dotted line shown in panel (a) of Figure S5 is considered to be the maximum possible blockade gap, then the *E*_C_ of this second diamond is estimated to be 23.3 meV. If instead, the maximum blockade gap is larger than what was observed, then the *E*_C_ is even larger since the maximum blockade gap equals four *E*_C_.

Table 1 lists the gate couplings (α), charging energies (*E*_C_), and total capacitances (*C*), extracted from the Coulomb diamond-like features for three devices in which three-terminal measurements on ferritin were executed. Similar values are obtained; in particular, similar ranges of *C* (and therefore of *E*_C_) are found (from 3.2 to 10.5 aF). In addition, the gate coupling (α) is much larger in these devices with the local-gate electrode (∼0.1 mean value), compared to the ones with the silicon-back gate previously reported (∼0.003 mean value^41^), confirming the improvement on α on the devices that include a local gate underneath the gap area.

**Table 1:** Summary of the Gate Coupling (α), Total Capacitance (*C*), and the Charging Energy (*E*_C_), Estimated From the Coulomb-Diamonds in the Stability Diagrams of Devices Ft1, Ft2, and Ft3

device	α	*C* (aF)	*E*_C_ (meV)
Ft1	0.0089	8.9	9.1
Ft2	0.0106	6.6	12.1
Ft3 (s)	0.2943	10.5	7.6
Ft3 (b)	0.0286	3.2	25.0

(s): parameters extracted from the small diamond shown in Figure S5a.

(b): average values associated with the large energy scale including the diamond-like features that are present in Figure S5 (yellow-dotted lines) and in Figure S7.

## Discussion

We first discuss the nonclosing Coulomb diamond structures as shown in Figure 4. First, the fact that the diamonds never close at zero bias indicates that the tunneling is through two or more islands that are connected in series.^40^ Second, the two observed different energy scales (23.3 meV vs 7.6 meV) suggest that two islands of different sizes are involved in the electron transport between the source and drain electrodes. A possible scenario that is consistent with our data is shown in Figure 4c, where the tunneling paths of an electron traveling from the source to the drain are indicated by the green arrows. First, instead of having an electron that tunnels directly to the ferritin core, the electron is assumed to tunnel from the source electrode to an iron atom (or a cluster of a few irons) that is located in a redox center within the organic shell (green circle) or near it. In the redox centers, Fe(II) is oxidized to Fe(III) before entering and becoming part of the mineral core, therefore, the presence of iron ions within the shell is feasible. Second, the electron tunnels from the iron ion or small cluster to the ferritin core. Third, the electron tunnels from the ferritin core to the drain electrode.

The second behavior, the closing diamonds, is visible in device Ft1 (Figure 3) and in device Ft2 (Figure S4). The fact that the diamond closes and that there is an excellent agreement between the Coulomb blockade simulations and the data (Figure 3), visible both in individual *IV*s [panels (a) and (b)] and in part of the stability diagram [panel (c)] indicates that charge transport is dominated by two-step electron tunneling: from the source electrode to the ferritin core, followed by the tunneling from the ferritin core to the drain electrode. However, this standard SET interpretation lacks an explanation for the blurred Coulomb blockade-like features that are observed as small fluctuations in the d*I*/d*V*, which seem to be stochastic, and intriguingly are also present in the stability diagram as a function of time, at zero gate voltage (Figure 3d, white arrows).

A possible scenario is presented in Figure 4c. First, the classic single-electron transport consisting of the tunneling from the source to the ferritin core, followed by the tunneling from the core to the drain, is indicated by the unidirectional red arrows. Second, the abrupt changes in the d*I*/d*V* are attributed to an electron that is tunneling back and forth onto an iron atom located within the ferritin shell (bidirectional red arrow), causing fluctuations in the electrostatic potential of the iron atom. The variation of the electrostatic potential in the iron atom produces changes in the chemical potential of the ferritin core, which causes the abrupt changes in the d*I*/d*V* of the ferritin, i.e., the iron atom acts as a small gate electrode.

The relation of the conductance (two-level) jumps or oscillations with the transfer of single electrons in a nearby island due to thermal excitation has also been described in refs (42 and 43), among others, albeit for different systems. As a first step in understanding the CB features in the data, the feasibility of having two particles of different sizes connected in series is further explored and described in the Supporting Information. Based on these estimates, it is more likely that the small island is attributed to a cluster of a few iron atoms. However, another possibility is to consider the small island to be assigned to a small iron aggregate inside the ferritin shell that is not connected to the main ferritin core. This hypothesis is supported by the transmission electron microscopy analysis on ferritin cores published by Pan et al.,^44^ according to which, depending on the iron loading, the different nucleation centers in the ferritin could sometimes remain apart from each other. We finally remark that we cannot exclude the possibility of two ferritin particles being responsible for the observed behavior. However, the presence of a small and large island makes this scenario less likely.

## Conclusions

The correspondence of the data shown in this article with those published in ref (36) validates the findings associated with the *IV* characteristics displaying, at low temperatures, Coulomb blockade behavior. Most importantly, we could modulate the charge transport through ferritin with a local gate. The fact that the three-terminal measurements are in good agreement with the Coulomb blockade theory allows the extraction of the relevant parameters, including the gate coupling. In cases where an individual SET is observed with closing diamonds of similar sizes, jumps in the differential conductance can be attributed to a gate effect caused by an electron tunneling in and out onto iron atoms within the ferritin shell or in a small cluster inside the ferritin, that is not in direct contact with the main ferritin core but causing changes in the ferritin chemical potential. The nonclosing diamonds of different sizes, on the other hand, indicate sequential tunneling events through more than one island in series. A possible scenario is that the electron tunnels from the source electrode to a small island (an iron atom or a small cluster of them), then to the main ferritin core, and last to the drain electrode. The appearance of one of these scenarios depends on the exact position of the ferritin particle in the gap and its composition, which can vary from particle to particle. Nevertheless, in all cases transport through a ferritin particle was shown to be gate-dependent thereby constituting a ferritin transistor. It would be of interest to further investigate the behavior of ferritin at low temperatures with different iron loads and to model the transport behavior in more detail.

## Supporting Information Available

The Supporting Information contains information regarding the device fabrication, the stability diagram acquired on device Ft2, the simulation of the stability diagram of device Ft1, and the capacitance estimates for the islands. Data supporting the findings of this paper are available in 4TU.ResearchData, see DOI: 10.4121/88bc422c-1b09-458e-a664-a6f209bb2a27 (PDF)
