# Supplementary material for: Ferritin Single-Electron Transistor
Source: J Phys Chem B. 2024 Jun 25;128(26):6387–93. doi: 10.1021/acs.jpcb.4c01937 (PMC11228996; doi:10.1021/acs.jpcb.4c01937)
Supplement: Supplementary file 1 — jp4c01937_si_001.pdf [file jp4c01937_si_001.pdf]

A Ferritin Single-Electron Transistor Supporting Information

Jacqueline A. Labra-Muñoz[†,‡] and Herre S. J. van der Zant[*,†]

†*Kavli Institute of Nanoscience, Delft University of Technology, 2628 CJ Delft, the Netherlands*

‡*Department of Physics, Huygens-Kamerlingh Onnes Laboratory, Leiden University, 2300 RA Leiden, the Netherlands*

E-mail: h.s.j.vanderzant@tudelft.nl

Contents

Chip design and device fabrication **S2**

Simulated stability diagram, based on device Ft1 **S4**

Stability diagram acquired on device Ft2 **S5**

Coulomb diamond on device Ft3 **S6**

Capacitance estimates **S7**

Additional three-terminal measurement on device Ft3 **S10**

Chip design and device fabrication

Platinum wide self-aligned nanogap devices were fabricated on Si/SiO_2 substrates. The spacing between the main electrode (ME) and auxiliary electrode (AE), from now on "the gap", is not defined by direct writing, but it results from a chemical process, in particular, from chromium oxidation. Figure S1a shows the chip design: In light blue, the ME (Ti/Pt of 5/30 nm); in dark blue, the 36 AE (Ti/Pt of 5/20 nm); in pink, the local gate electrode (Ti/Pt of 3/13 nm), which is separated from the source-drain electrodes by an insulating hafnium oxide layer (22 nm). The white numbers indicate the labeling order of the devices. Figure S1c shows the schematic of one "device", which is the assemblage formed by a pair of ME-AE (depicted as source-drain, respectively), the gap in between them, and the local back gate electrode (GE) below them. The gap length (size) is defined as the shortest distance between ME and AE (red-dotted line, Fig. S1c). The gap width is the projection of the AE on the ME (black dotted line), which is 1 μm.

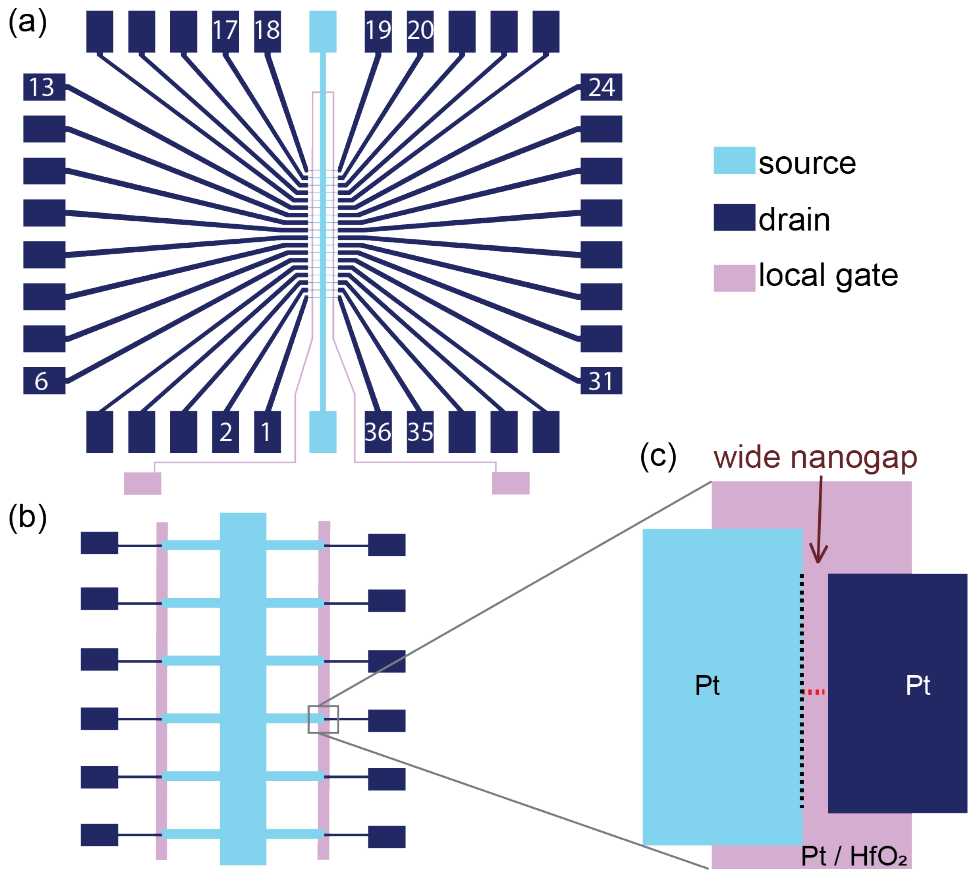

Figure S1: Wide self-aligned nanogap chip design. (a) General overview. In light blue: the main electrode (depicted as the source). In dark blue: the auxiliary electrodes (represented as the drain). In pink: the local gate electrode. (b) Close-up of the center of the chip. (c) Schematic of one "device", formed by the gap, source-drain electrodes, and gate electrode. The gap length/size (8-33 nm) is represented by the red-dotted line. The black-dotted line indicates the gap width ($\sim$ 1 μm).

The fabrication of the devices is summarized in Fig. S2. On top of a doped Si/SiO_2 substrate, the local back gate electrode (GE) is defined by e-beam lithography (EBL) and evaporation of 3 nm of titanium and subsequently of 13 nm of platinum (Fig. S2a-e). The GE is then covered with hafnium oxide deposited by atomic layer deposition (ALD) (Fig. S2f). A second EBL cycle defines the ME, by depositing 5/30 nm of titanium/platinum and $20-25$ nm of chromium (Fig. S2g). Upon oxidation in an oxygen plasma, chromium expands its size. In this manner, chromium oxide acts as a shadow mask of a few nanometers near the edge of the first electrode (Fig. S2h). The thickness of the chromium layer and its exposure to oxygen plasma determine the expansion and, thus, the size of the gap. A third EBL cycle

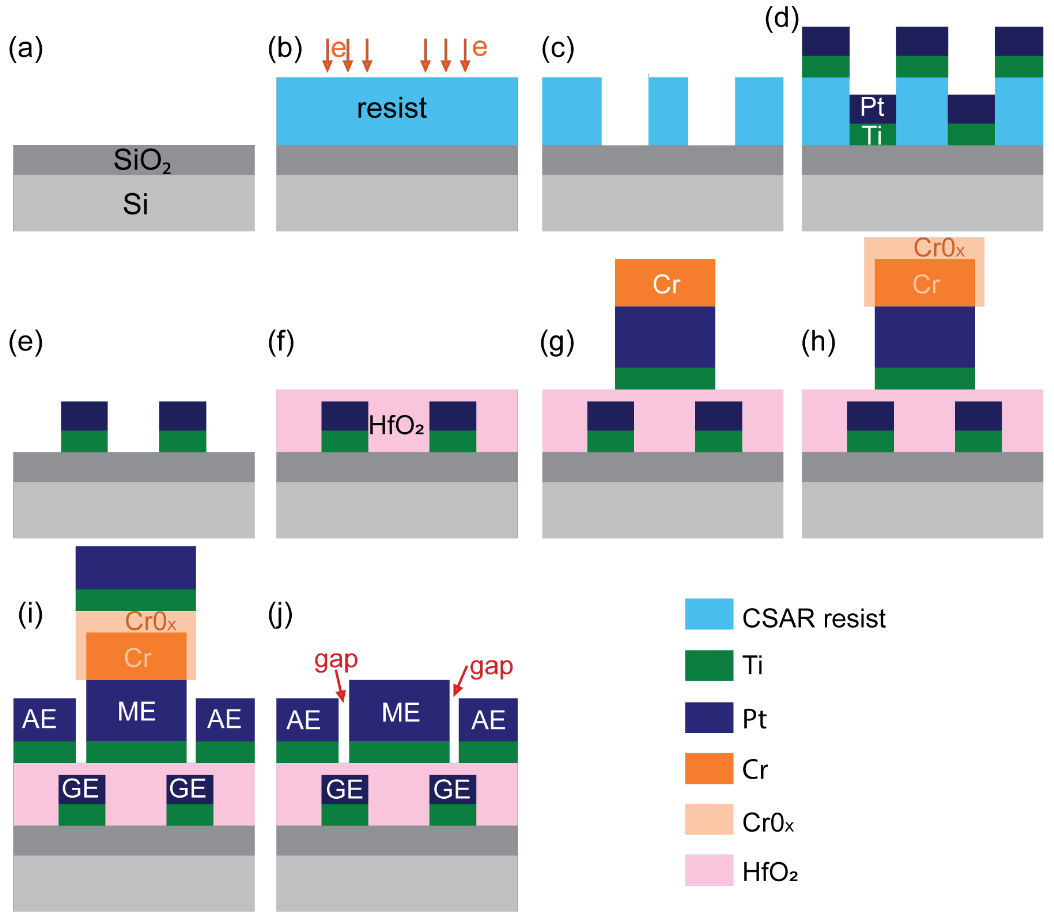

Figure S2: Schematic of the fabrication process. (a) Chemical cleaning of a doped Si/SiO_2 wafer. (b) Spin coating and baking of CSAR AR6200 positive resist, followed by e-beam exposure to define the GE. (c) Development in pentyl acetate (1 min.) and wet resist descum (5 s in xylene). (d) Evaporation of Ti/Pt (3/13 nm). (e) Lift-off. (f) ALD of hafnium oxide ($\sim$22 nm). (g) Second e-beam lithography cycle to define the ME. The evaporation consists of Ti/Pt/Cr (5/30/25 nm). (h) Chromium oxidation by oxygen plasma, defining the gap size. (i) Third e-beam lithography cycle to define the AE. The evaporation consists of Ti/Pt (5/20 nm). (j) Chromium wet-etching (1.5 min.) followed by oxygen plasma for cleaning. The devices are finished.

defines the AE, with the deposition of 5 nm of titanium and 20 nm of platinum (Fig. S2i). In the final step, the chromium layer is wet-etched away to reveal the underlying nanogaps (Fig. S2j).

Simulated stability diagram, based on device Ft1

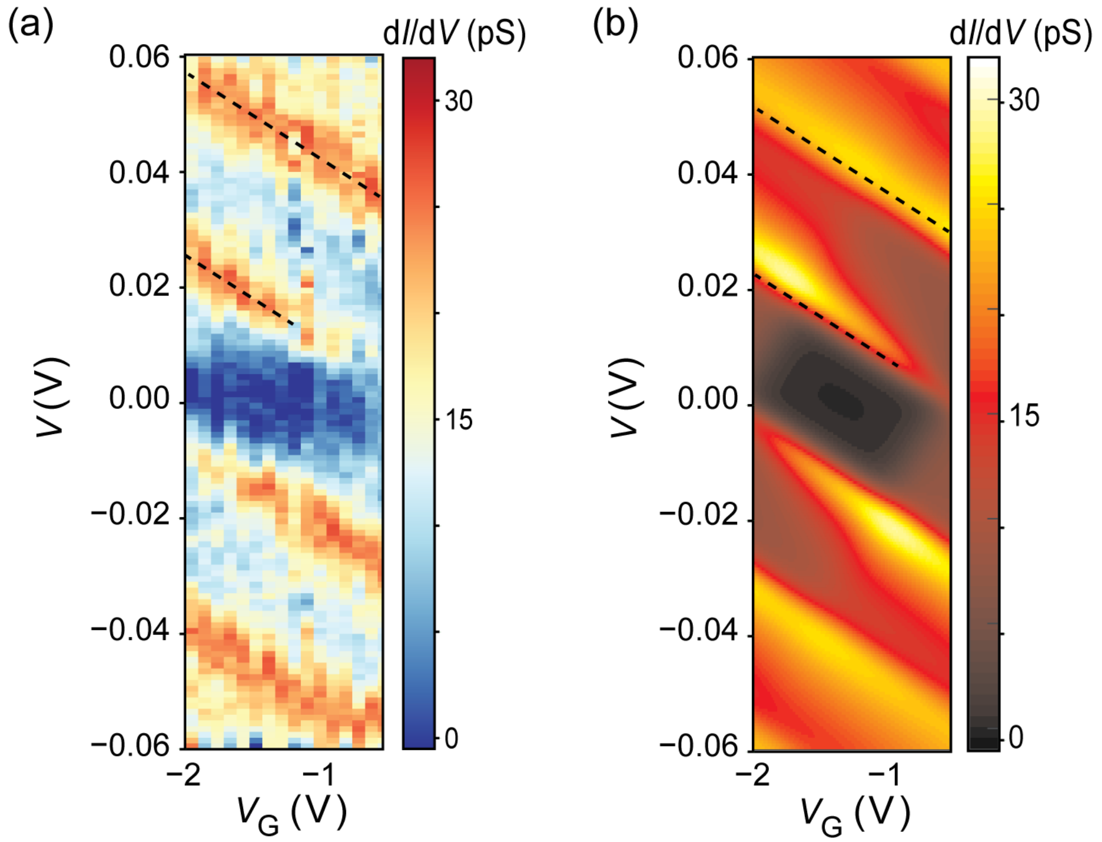

Figure S3: Stability diagrams. (a) Three-terminal measurement acquired on device Ft1 at 10 K, while sweeping V from negative to positive values. (b) Simulated stability diagram for device Ft1. The parameters used for the simulation are: $C_1 = 6.0$ aF, $C_2 = 2.7$ aF, $R_1 = 34$ GΩ, $R_2 = 14$ GΩ, and $C_G = 0.1$ aF, $T = 10$ K; the gate coupling α ($= C_G/C$) is 0.009.

Table S1: Charging energy (E_C), total capacitance (C), left (source) and right (drain) capacitances (C_1 and C_2), and the gate capacitance (C_G) obtained from the Coulomb diamond observed in the stability diagram of device Ft1 (Fig. 3 from main article).

Parameter	E_C (meV)	C (aF)	C_1 (aF)	C_2 (aF)	C_G (aF)
Experimental CB diamond	9.05	8.85	5.65	3.12	0.08

Stability diagram acquired on device Ft2

Figure S4a shows the stability diagram recorded on device Ft2, at 10 K. As in device Ft1, a Coulomb blockade diamond is visible, indicated by the black-dotted lines as a guide to the eye. From the parameters β and γ, α was estimated to be 0.01. From the height of the diamond, an estimated $E_C = 12$ meV was obtained. This stability diagram was generated with IVs recorded while sweeping V from positive to negative values. Note, that the values of α and E_C are similar to those found for device Ft1. The red and yellow dotted lines indicate the differential conductance of the two IV's that are displayed in panel (b), at gate voltages of -1.5 and -2.5 V, respectively, denoting that the transport is modulated by changing the gate voltage. At voltages larger than 20 mV, the resistance is estimated to be ~ 5.4 GΩ, which is in agreement with the resistances previously reported for ferritin particles.[1]

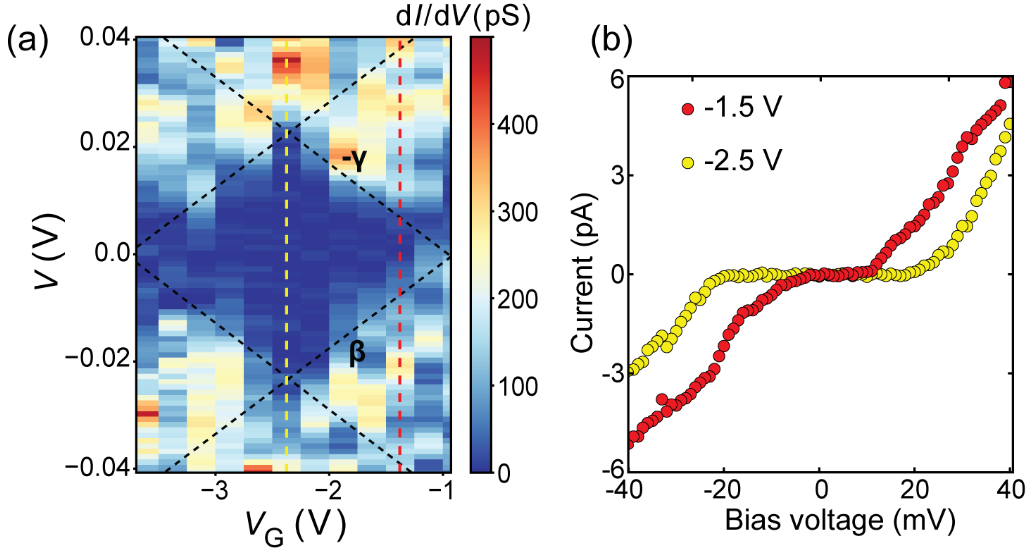

Figure S4: Three-terminal measurements on device Ft2, at 10 K. (a) Stability diagram generated with the IVs recorded while sweeping V from positive to negative values. The black-dotted lines indicate the slopes of the Coulomb diamond: $\beta = 0.0188$, $\gamma = 0.0241$. From the slopes and the height of the diamond, α was estimated to be 0.0106, $C = 6.6$ aF, $E_C = 12.1$ meV, $C_1 = 3.7$ aF, $C_2 = 2.8$ aF and $C_G = 0.07$ aF. The yellow and red dotted lines indicate the corresponding differential conductance of the IV's that are displayed in panel (b).

Coulomb diamond on device Ft3

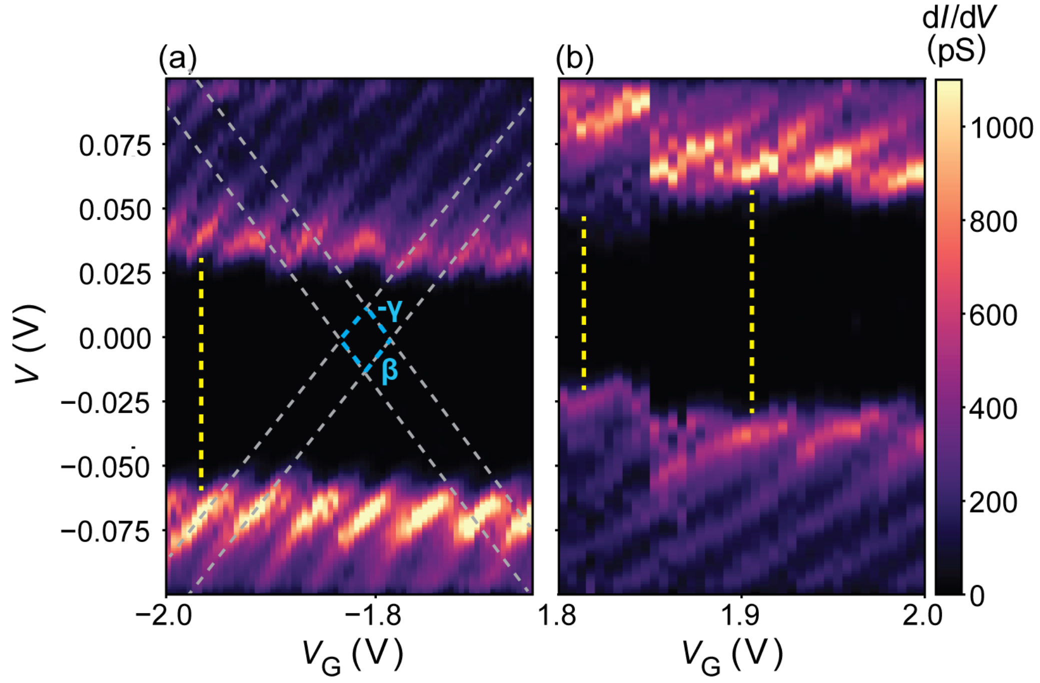

Figure S5: Experimental stability diagrams exhibiting non-closing Coulomb blockade diamonds, acquired on device Ft3 at 10 K, while V is swept from positive to negative values. (a) At a negative gate voltage range. (b) At a positive gate voltage range. In both panels, two energy scales are visible: The larger one is associated with the large blockade gap indicated by the yellow-dotted lines with observed minimum charging energy (E_C) of 23.3 meV. A smaller energy scale is observed ($E_C \sim 7.6$ meV), which is associated with the small Coulomb diamond-like structures that are indicated by the gray-dotted lines as a guide to the eye. One of these small diamonds is enclosed by the blue-dotted lines, whose slopes are estimated to be $\beta = 0.571$ and $\gamma = 0.607$. The estimated gate coupling α is 0.29

Capacitance estimates

To explore the feasibility of having two particles of different sizes connected in series, capacitance estimates are performed and compared with the experimental values acquired on device Ft3, which are displayed in Fig. S5 (and Figure 4a,b from the main article). The particle of smallest E_C (7.6 meV) and therefore the largest C (10.5 aF) can be attributed to the ferritin core, based on the capacitance estimates previously described,[1] in which $C = 11.3$ aF was estimated for the situation in which the ferritin particle was symmetrically connected to the source and drain electrodes and the contact area was maximized. On the other hand, the particle of smallest C (3.4 aF) from Fig. S5 is on the lowest size of the published capacitance estimates;[1] however, these estimates did not consider the possibility of having particles in series.

In the following, capacitance estimates of the small island are pursued, assuming that the island is formed by one or more iron atoms located at the external border of the ferritin shell (redox center). In the scheme of Fig. S6, the smallest island is indicated by the green circle, located in the lowest half of the ferritin shell, closer to the left (source) electrode. The parameter d_{vac} is the distance between the smallest island and the dielectric, and d_{haf} is the thickness of the dielectric (hafnium oxide, ~ 22 nm). In this simplified scenario, three capacitances that are connected between the small island and the source ($C_{\mathrm{Fe}_{\mathrm{source}}}$), ferritin core ($C_{\mathrm{Fe}_{\mathrm{core}}}$), and gate($C_{\mathrm{Fe}_{\mathrm{G}}}$) electrodes, contribute to the total small island's capacitance according to $C_{\mathrm{Fe}} = C_{\mathrm{Fe}_{\mathrm{source}}} + C_{\mathrm{Fe}_{\mathrm{core}}} + C_{\mathrm{Fe}_{\mathrm{G}}}$. Each of the three capacitances is modeled as the capacitance of a conductive sphere of radius r placed at a distance d from a metal plate, according to[2]

$$C = 4\pi\epsilon r \sum_{i=0}^{\infty} \frac{2 \cdot \sinh(\eta_0)}{e^{(1+2i)\eta_0} - 1}, \tag{1}$$

with $\eta_0 = \mathrm{acosh}(1 + d/r)$. A simplified expression for Eq. 1 is given in:[2]

$$C = 4\pi\epsilon r[1 + 0.5\log(1 + r/d)], \tag{2}$$

with $\epsilon = \epsilon_r \cdot \epsilon_0$ the permittivity of the medium and ϵ_r the relative permittivity of the medium. Based on the geometry described above, the distance between the small island and the source electrode is considered to be 1 nm, and the medium is vacuum ($\epsilon = \epsilon_0$). The distance between the small island and the ferritin core is determined by the ferritin shell thickness, i.e., 2 nm; in this case, since ferritin is the only medium involved, $\epsilon = \epsilon_{\mathrm{Ft}} \cdot \epsilon_0$ with ϵ_{Ft} the ferritin relative permittivity. The total $C_{\mathrm{Fe}_{\mathrm{G}}}$ includes two different mediums (vacuum and the dielectric), which can be seen as the equivalent capacitance of two capacitors connected in parallel, that differ on d and ϵ. Thus, $C_{\mathrm{Fe}_{\mathrm{G}}} = (C^{-1}_{\mathrm{Fe}_{\mathrm{G}}:\,\mathrm{dielec}} + C^{-1}_{\mathrm{Fe}_{\mathrm{G}}:\,\mathrm{vac}})^{-1}$, being the capacitances between the small island and the local gate in the dielectric and in vacuum, respectively.

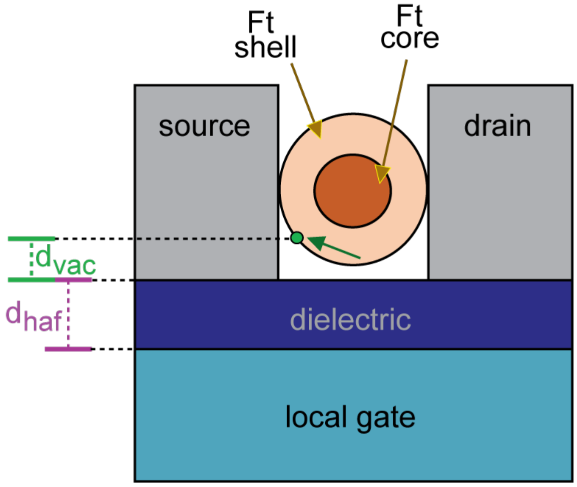

Figure S6: Schematic of the capacitance of the small island (green circle), formed by one or more Fe atoms. The estimates are based on the scenario in which successive tunneling between two islands of different sizes is expected. The distance between the small island and the dielectric layer is indicated by d_{vac}. The thickness of the dielectric (hafnium oxide) is represented by d_{haf}.

Table S2 displays a summary of the total capacitance estimates for a sphere composed of 1, 3, 5, and 7 iron atoms (C_{Fe}), as a function of the ferritin relative permittivity. It is considered that the diameter of one iron atom is 126 pm, a hafnium oxide relative permittivity of 15.5, $d_{vac} = 1$ nm, and $d_{haf} = 22$ nm. Based on these estimates, the small island is not likely to be attributed to one single iron ion, however, the experimental $C = 3.4$ aF can be ascribed to 3 or 5 iron ions, depending on the permittivity of the ferritin shell.

Another possibility is that instead of considering the island to be assigned to iron ions in the redox center, the small island could be a small iron aggregate inside the ferritin shell that is not connected to the main ferritin core. This last hypothesis can be sustained based on transmission electron microscopy (TEM) studies performed by Pan *et al.*,[3] on ferritin cores. Depending on the iron loading, the authors of that studu found that the different nucleation centers could sometimes remain apart from each other, i.e., not collapsing into one main nanoparticle.

The analysis presented above is just a first step in understanding the CB features in the data. More detailed capacitance calculations would be helpful, for example, using a finite element package such as Comsol. These calculations should also consider the mutual capacitance between the two islands, as to explain the size of the differential conductance jumps observed in the experiments.

Table S2: Charging energy ($E_{C_{Fe}}$), total capacitance (C_{Fe}), and gate coupling parameter (α_{Fe}) estimated for an island assumed to be composed of one (or more) Fe atom(s), located in the external side of the ferritin shell, closer to the source electrode (green circle depicted in Fig. S6); as a function of the relative permittivity of the ferritin shell (ϵ_{Ft}).

ϵ_{Ft}	# Fe atoms	$E_{C_{Fe}}$ (meV)	C_{Fe} (aF)	α
	1	256.8	0.31	0.042
20	3	83.5	0.96	0.043
	5	49.0	1.63	0.043
	7	34.4	2.33	0.043
	1	99.0	0.81	0.016
55	3	32.2	2.49	0.016
	5	18.9	4.23	0.016
	7	13.3	6.02	0.016

Additional three-terminal measurement on device Ft3

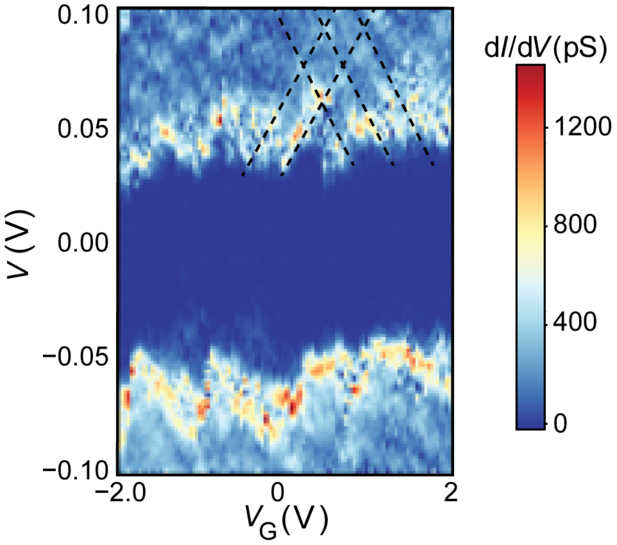

Figure S7: Experimental stability diagram measured on device Ft3 after ferritin deposition, at 10 K. Coulomb diamond-like structures are indicated by the black-dotted lines as a guide to the eye. The slopes are estimated to be $\beta = 0.048$ and $\gamma = 0.069$; α is 0.028.

References

(1) Labra-Muñoz, J. A.; de Reuver, A.; Koeleman, F.; Huber, M.; van der Zant, H. S. J. Ferritin-Based Single-Electron Devices. *Biomolecules* **2022**, *12*, 705.

(2) Riba, J.-R.; Capelli, F. Analysis of Capacitance to Ground Formulas for Different High-Voltage Electrodes. *Energies* **2018**, *11*, 1090.

(3) 3D morphology of the human hepatic ferritin mineral core: New evidence for a sub-unit structure revealed by single particle analysis of HAADF-STEM images. *Journal of Structural Biology* **2009**, *166*, 22–31.
